# Calibration of a multi-anvil high-pressure apparatus to simulate planetary interior conditions

**DOI:** 10.1140/epjti/s40485-018-0047-z

**Published:** 2018-07-04

**Authors:** J. S. Knibbe, S. M. Luginbühl, R. Stoevelaar, W. van der Plas, D. M. van Harlingen, N. Rai, E. S. Steenstra, R. van de Geer, W. van Westrenen

**Affiliations:** 1 Faculty of Science, Vrije Universiteit Amsterdam, Amsterdam, the Netherlands; 2 Department of Earth Sciences, Indian Institute of Technology Roorkee, Roorkee, India

**Keywords:** High pressure, Multi-anvil, Experimental petrology, Planetary interiors

## Abstract

This paper presents the setup and pressure calibration of an 800-ton multi-anvil apparatus installed at the Vrije Universiteit (Amsterdam, the Netherlands) to simulate pressure-temperature conditions in planetary interiors. This high-pressure device can expose cubic millimeter sized samples to near-hydrostatic pressures up to ~ 10 GPa and temperatures exceeding 2100 °C. The apparatus is part of the Distributed Planetary Simulation Facility (DPSF) of the EU Europlanet 2020 Research Infrastructure, and significantly extends the pressure-temperature range that is available through international access to this facility.

## Introduction

A fundamental problem hampering studies of the composition and structure of the terrestrial planets is that most of their mass is located at depths inaccessible to direct investigation. The occurrence and behavior of solid and molten silicate and metal phases at ambient pressures are relatively well constrained due to analyses of natural surface samples. For Earth, some additional insights into the chemical properties of the deeper subsurface can be obtained indirectly from analyses of natural samples of deep origin, such as extrusive volcanic rocks formed by partial melting of planetary mantles and magmatic minerals from deeper levels entrained in such rocks during their transport to the surface (e.g., [1–4]), high-pressure metamorphic rocks formed at great depths but subsequently exhumed to the surface in mountain belts (e.g. [5]), and the study of mineral inclusions in diamonds formed hundreds of kilometers underneath the surface (e.g., [3]). Seismology can also provide a window into the physical and chemical properties of planetary interiors but is currently limited to studies of the Earth (e.g. [6–8]) and the Moon (e.g. [9, 10]). Interior models for other planets and moons in the solar system are constrained by remote sensing and geodesic data obtained by space missions (e.g., [11–14]).

Correct interpretation of measured planetary characteristics for interior models relies on the understanding of high-pressure and high-temperature phase mineralogy. With the use of physical and chemical laws which govern the crystal structure of minerals, e.g. thermodynamic relations and density functional theory, ambient-condition crystalline properties can be extrapolated to high pressures and high-pressure phases can be predicted (e.g. [15, 16]). However, such methods become computationally expensive and impractical for systems containing numerous elements, and have difficulties in predicting material properties in the high-temperature regime of deep planetary interiors.

Experimental techniques that expose samples to high pressures and temperatures provide crucial complementary constraints on the matter of planetary interiors. For interior pressures to a maximum of approximately 3.5-4 GPa, a so-called piston cylinder press can be used to compress cubic millimeter sized natural or synthetic rock samples while simultaneously heating the samples to a maximum of approximately 1650 °C (e.g. [17]). Two such piston-cylinder apparati at Vrije Universiteit Amsterdam, the Netherlands, are currently part of the Distributed Planetary Simulation Facility (DPSF) of the EU Europlanet 2020 Research Infrastructure, providing access to European planetary scientists interested in using this technique. Facilities that can achieve higher pressures relevant for Earth’s upper mantle and the deep interiors of the Moon, Mercury, Mars and Ganymede would help expand the opportunities of this scientific community.

Here, we present the experimental setup and assembly pressure calibration of an 800 ton multi-anvil apparatus at the Vrije Universiteit Amsterdam, the Netherlands. This press can expose cubic millimeter-sized samples to pressures up to ~ 10 GPa and temperatures up to ~ 2100 °C with the assembly used in this study and significantly extends the pressure-temperature range that is available through international access to this facility.

Since the first introduction of multi-anvil presses more than half a century ago, developments of this type of presses have significantly improved their experimental reproducibility, extended the attainable pressure and temperature range, and hence expanded the corresponding research opportunities [18]. Although multi-anvil apparati are widely used for examining the physical and chemical characteristics of planetary materials, details of their calibration are rarely published, hampering full comparison of results obtained at different facilities. Here, we present full details of the calibration of the device, including an assessment of uncertainties.

## Methods/Experimental

### Experimental setup

**Fig. 1:**
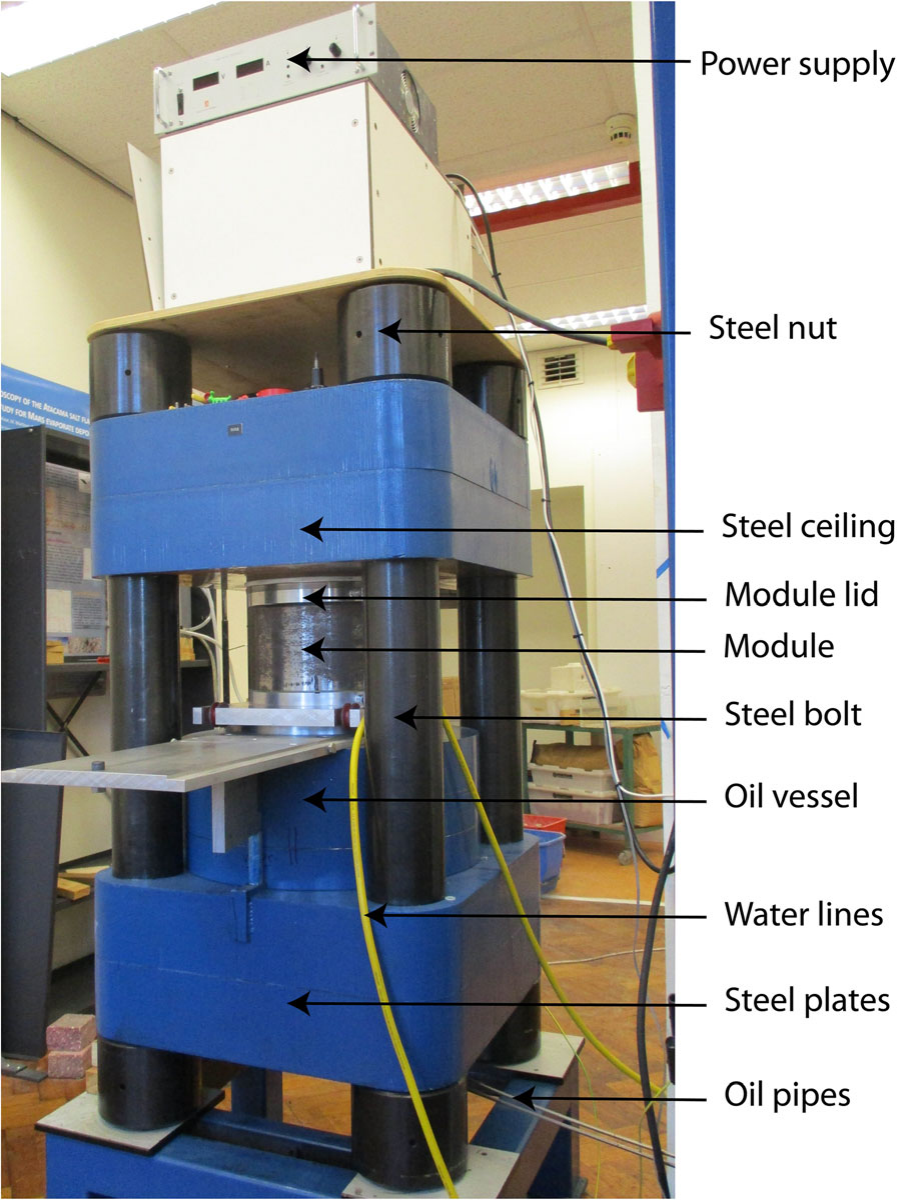
The multi-anvil apparatus at Vrije Universiteit Amsterdam

**Fig. 2:**
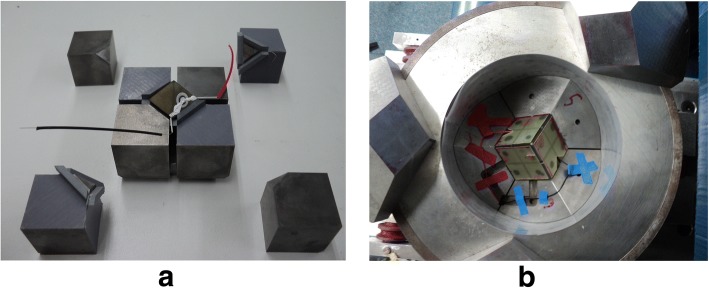
The experimental setup: **a** the assembly within the eight tungsten inner anvils. **b** The pressure module with outer anvils surrounding the cube of inner anvils

The 800 ton hydraulic press was built by the workshop of the Department of Earth Sciences at the University of Bristol, UK. The apparatus contains a Walker-type pressure module [19] with a 15 cm tall, 20 cm inner diameter, and 6.3 cm thick hardened steel wall that rests on an oil vessel. The module holds anvils in a Kawai geometry [20], consisting of six hardened steel outer wedges and eight cubic-inch-sized tungsten carbide (WC) inner anvils (grade THM-U, Kennametal, Arnhem, the Netherlands). A hardened steel module lid rests on the top three wedges and closes the module. An hydraulic pumping system regulates the pressure in the oil vessel, which controls the pressure in the module generated by pressing the module upward against a steel ceiling (Fig. 1). Mylar sheets (0.1 mm thickness), lubricated with polytetrafluorethylene (PTFE) spray, line the inner wall of the module and the outer surfaces of the outer wedges to reduce friction and prevent electrical conductance between the wedges and the module wall [19]. All inner anvils have one truncated corner with 11 mm edge length (TEL = truncated edge length = 11 mm). They are cubically assembled and enclose an octahedral sample assembly with 18 mm edge lengths (OEL = octahedron edge length = 18 mm), leaving ~ 3.4 mm space in between the anvils (Fig. 2). Pyrophyllite gaskets of 3.3/3.0 mm height/width are placed between the inner anvils near the truncated edges to prevent contact between inner anvils and prohibit outward flow of the sample assembly’s pressure medium during compression. PTFE tape is placed snugly against the back of the gaskets to minimize extrusion of these gaskets during compression. The cubically assembled inner anvils are covered and held together by sheets of epoxy resin fiberglass (known as G10 sheets, 0.65 mm thickness) glued on each side of the cubic assemble. These G10 sheets also prevent electrical conductance between the inner anvils and the outer wedges. Two copper foils are placed through cuts in the G10 sheets and electrically connect the bottom inner anvil with one of the lower wedges and the top inner anvil with one of the upper wedges. These yield electrically connected paths from the bottom octahedral surface to the module bottom plate and from the top octahedral surface to the module top plate, via the inner anvils, copper and wedges.

**Fig. 3:**
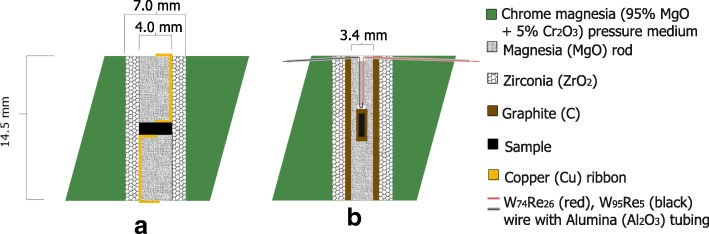
Schematic assembly setup for the performed calibration experiments. **a** The 18/11 OEL/TEL assembly for room temperature calibration experiments. **b** The 18/11 OEL/TEL assembly for high temperature calibration experiments

The sample assembly (Fig. 3) is a Cr_2_O_3_-doped MgO octahedron (95% MgO and 5% Cr_2_O_3_ with 30% porosity, from Japan Ceramic Engineering Co., Ltd.), with a 7 mm diameter central hole [21]. A zirconia (ZrO_2_) sleeve of 4 mm inner diameter (drilled out of plates from Mino Yogyo Ceramics, Japan) is placed in the hole and acts as heat insulator for its interior. The assembly set-up interior to the ZrO_2_ sleeve varies depending on the type of experiment. In this study, sample pressures are calibrated with respect to the vessel’s oil pressure by identifying and bracketing known high-pressure phase transitions. Electrical resistance measurements at room temperature are performed to detect high-pressure transitions in metals in situ. High-temperature quench (ex situ) experiments are performed to bracket high-pressure phase transitions in silica (SiO_2_) and in calcium germinate (CaGeO_3_).

For experiments that measure electrical resistance of a sample at room temperature, the ZrO_2_ sleeve contains two MgO rods that squeeze a sample of thin needle-like dimensions at the assembly’s centre. Two copper ribbons are placed along the outside of the MgO rods to electrically connect the sample with the two opposite surfaces of the octahedral assembly and close the electrical path from the top to the bottom of the module trough the sample (Fig. 3a). An ohmmeter measures the electrical resistance between the top and bottom of the module in situ during sample compression.

For high-temperature quench (ex situ) experiments, a graphite sleeve with 3.6 mm inner diameter is placed inside the zirconia and closes the electrical path from the top of the module to the bottom. A 2 mm long graphite sample bucket with a 1 mm thick lid contains the sample powder and is placed in the middle of the assembly, encapsulated by crushable polycrystalline MgO parts (Fig. 3b). Type C (W_95_Re_5_ and W_74_Re_26_) thermocouple wires are guided through alumina tubes to the sample and create a junction just above the sample container. An electrical voltage is applied by a DC power supply (Delta Elektronica) between the top and bottom of the module to heat the sample environment by the ohmic dissipation in the graphite cylinder. The temperature difference between the thermocouple junction and the ends of the thermocouple wires induces a voltage at the thermocouple ends (the Seebeck effect). We measure this voltage and relate it to sample temperature using the ASTM E230/E230M standard from the National Institute of Standards and Technology (NIST).

### Starting materials and phase transitions

Room temperature resistance measurements are performed on metallic bismuth (Bi) samples (Alfa Aesar, 99.999% purity). Bismuth phase transitions from trigonal to monoclinic (Bi I-II) at 2.55 ± 0.006 GPa, from monoclinic to tetragonal (Bi II-III) at 2.69 ± 0.01 GPa, and from tetragonal to body-centered cubic (Bi III-V) at 7.66 ± 0.18 GPa are historically well studied by a variety of methods including detecting changes in sample volume, shock measurements, detecting variations in electrical resistance and by crystal structure measurements using in-situ X-ray diffraction ([22]; and references therein). We identify the phase transitions by detecting characteristic variations in the measured electrical resistance during compression.

High temperature quench experiments are performed with CaGeO_3_ (synthesized to wollastonite structure) and hydrated SiO_2_ (Qtz structure) (Alfa Aesar) sample powders. At 1200 °C, a phase transition in SiO_2_ from trigonal (α-) quartz to monoclinic coesite takes place at 3.2 ± 0.1 GPa [23–29]. At 1300 °C, a phase transition from monoclinic coesite to tetragonal stishovite takes place in SiO_2_ at 9.4 ± 0.4 GPa [23, 29–35]. At 1000 °C, a phase transition in CaGeO_3_ from tetragonal garnet to orthorhombic perovskite occurs at 6.2 ± 0.2 GPa [36–41].

### Experimental procedure and sample analysis

After loading the sample assembly, pressure is increased at a rate of 20 bar per hour for high-temperature experiments and 10 bar per hour for room temperature experiments. For the latter, electrical resistance is measured continuously during this compression stage. After the target pressure is attained, high-temperature experiments are heated at a rate of 50 °C per minute to the target temperature. Temperature is held constant at the target value for a minimum of 30 min and up to a few hours. Samples are quenched by switching off the electrical power. When the experiment is finished, i.e. after target pressure is achieved for a room temperature experiment or when the module is cooled down to below 50 °C after a high-temperature experiment, pressure is reduced by 10 bar per hour to ambient conditions.

Quenched samples are embedded in a 1 in. epoxy mount and ground with alumina paper until the sample surface is exposed. We collected Raman spectra from these samples to determine the sample’s mineralogy using a red (785 nm) laser on a Renishaw InVia Reflex confocal Raman microscope at Vrije Universiteit Amsterdam with a grating of 1200 grooves/mm.

## Results

### Sample analysis

**Fig. 4:**
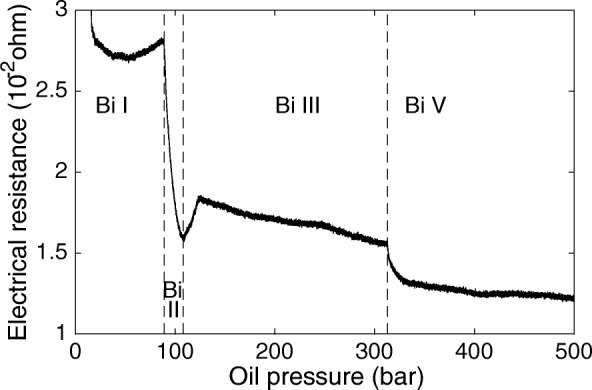
Electrical resistance of bismuth (Bi) measured in-situ during a high pressure experiment at room temperature

The electrical resistance measured in-situ through a Bi sample during a room temperature, high-pressure experiment is plotted in Fig. 4. The detected variations in electrical resistance identify the Bi I-II phase transition at an oil pressure of 89 bar, the Bi II-III phase transition at an oil pressure of 108 bar, and the Bi III-V phase transition at an oil pressure of 313 bar. These transitions are used as a first-order indication for the relation between sample pressure and oil pressure at high temperatures.

**Fig. 5:**
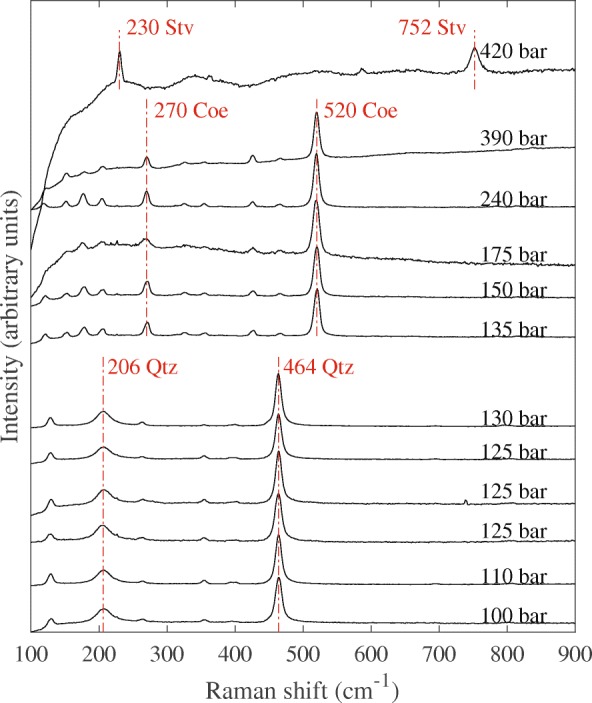
The Raman spectra from recovered SiO_2_ samples obtained from the experiments listed in Table 1. Characteristic peaks of quartz, coesite and stishovite are labelled by the abbreviations ‘Qtz’, ‘Coe’ and ‘Stv’, respectively

Raman spectra of quenched SiO_2_ samples, obtained from high-temperature quench experiments listed in Table 1, are shown in Fig. 5. Experiments MA16 and MA11 with oil pressures at 130 bar and 135 bar bracket the disappearance of quartz peaks at ~ 206 cm^− 1^ and ~ 464 cm^− 1^ and the appearance of coesite peaks at ~ 270 cm^− 1^ and ~ 520 cm^− 1^. Experiments MA31 and MA33 with oil pressures at 390 bar and 420 bar bracket the disappearance of ~ 270 cm^− 1^ and ~ 520 cm^− 1^ peaks and the appearance of stishovite peaks at ~ 230 cm^− 1^ and ~ 752 cm^− 1^.

**Table 1:** ^a^Thermocouple readout failed in these experiments. These temperatures have been estimated based on the power-temperature relationship of other experiments at similar pressures and carry an uncertainty of ±150 degrees

Experiment	Starting composition	Oil pressure (bar)	Power (W)	Temperature (°C)	Duration (hr:min)	Resulting polymorph
MA2	SiO_2_	240	700	1100^a^	3:00	Coe
MA4	SiO_2_	300	500	750^a^	2:50	Coe
MA5	SiO_2_	175	500	750^a^	3:30	Coe
MA6	SiO_2_	175	844	1100	1:50	Coe
MA7	SiO_2_	150	773	1200	3:30	Coe
MA8	SiO_2_	100	805	1200^a^	2:00	Qtz
MA9	SiO_2_	125	755	1200^a^	1:00	Qtz
MA11	SiO_2_	135	766	1200	3:30	Coe
MA13	SiO_2_	110	682	1200	5:00	Qtz
MA14	SiO_2_	125	665	1200^a^	2:00	Qtz
MA15	SiO_2_	125	674	1200	2:00	Qtz
MA16	SiO_2_	130	717	1200	6:00	Qtz
MA23	CaGeO_3_	260	574	950^a^	1:30	Grt + Prv
MA24	CaGeO_3_	300	695	1000^a^	1:35	Prv
MA25	CaGeO_3_	280	667	1000^a^	1:10	Prv
MA26	CaGeO_3_	270	742	1000	1:20	Prv
MA27	CaGeO_3_	265	651	1000	1:30	Prv
MA28	CaGeO_3_	260	895	1000	1:00	Prv
MA29	CaGeO_3_	263	650	1000^a^	1:00	Prv
MA30	CaGeO_3_	255	670	1000^a^	1:05	Grt
MA31	SiO_2_	390	982	1300	0:30	Coe
MA33	SiO_2_	420	878	1300	1:00	Stv

**Fig. 6:**
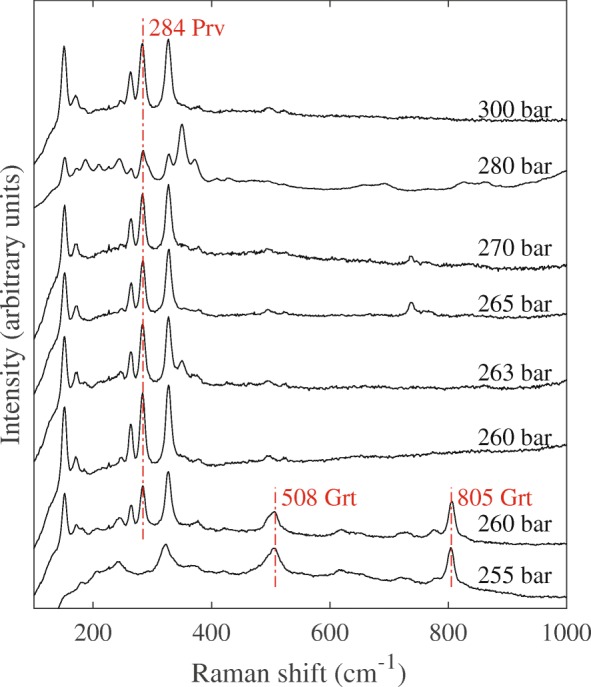
The Raman spectra from recovered CaGeO_3_ samples obtained from the experiments listed in Table 1. Characteristic peaks of garnet-structured and perovskite-structured polymorphs are labelled by the abbreviations ‘Grt’ and ‘Prv’, respectively

Raman spectra of quenched CaGeO_3_ samples, obtained from high-temperature quench experiments listed in Table 1, are shown in Fig. 6. Both experiments MA30 at 255 GPa and MA23 at 260 GPa show clear garnet-structured peaks at 508 cm^− 1^ and 805 cm^− 1^ [40], which are absent in samples that were quenched at higher pressure. The perovskite-structure peak at 284 cm^− 1^ [42] is present in MA23 and all samples that were quenched at higher pressure. The Raman spectrum of MA23 contains both the garnet and perovskite structure signatures and marks the location of the phase transition. Sample MA28, also obtained from an experiment at 260 bar, does not show any Raman signature for garnet. We go into more detail on this issue in the discussion section.

### Calibration curve

**Fig. 7:**
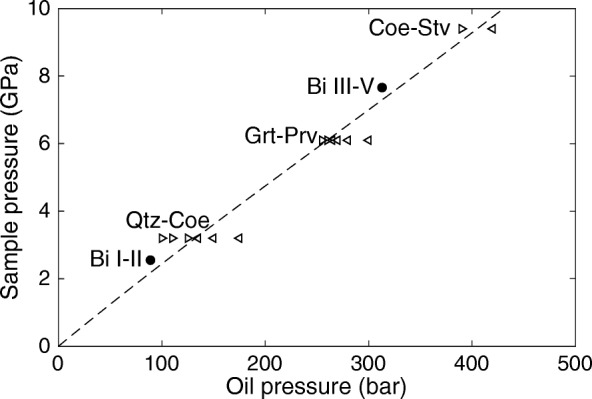
The pressure calibration curve which relates the oil pressure in the oil vessel to the attained sample pressure for temperatures at room temperature (Bi) and at ~ 1300 °C. The dashed line is obtained from a spline interpolation through the high-temperature calibration points

We obtain a pressure calibration curve by spline interpolation. The Bi phase transitions are not used in the interpolation, because they have been identified at room temperature whereas we are interested in the pressure calibration at high temperatures. Figure 7 plots the pressure calibration curve, of which Table 2 lists consecutive 0.5 GPa sample pressure points.

**Table 2:** A list of points on the calibration curve

Oil pressure (bar)	Sample pressure (GPa)
21	0.5
41	1
62	1.5
83	2
104	2.5
125	3
146	3.5
168	4
190	4.5
212	5
234	5.5
257	6
279	6.5
301	7
323	7.5
345	8
367	8.5
389	9
410	9.5
431	10

## Discussion

### Temperature errors

The temperature distribution in high-pressure assemblies is known to be heterogeneous in axial and radial dimensions. In our experiments, temperature is measured by a thermocouple at the wire-junction just outside the graphite sample container, approximately 1.5 mm away from the center of the assembly and sample. For an indication on the temperature difference between the sample and the thermocouple junction as well as the extent of thermal heterogeneity, we have modelled the temperature distribution in our assembly using the numerical method of Hernlund et al. [43]. The temperature distribution in the assembly is assumed to be axisymmetric about the central axis of the assembly (the *r* = 0 line) and symmetric about the assembly’s horizontal mid-plane (the z = 0 plane), where zero heat flux boundary conditions are applied. Constant temperature boundary conditions are applied at z = 11 mm and *r* = 11 mm of un-prescribed value, which is interior to the WC cubes and far from the assembly’s center. We used the model setting that accounts for the effect of the surrounding module, which prescribes a constant boundary temperature of 25 °C several hundreds of millimetres away from the sample [43]. A thermocouple temperature of 1200 °C was assumed.

**Fig. 8:**
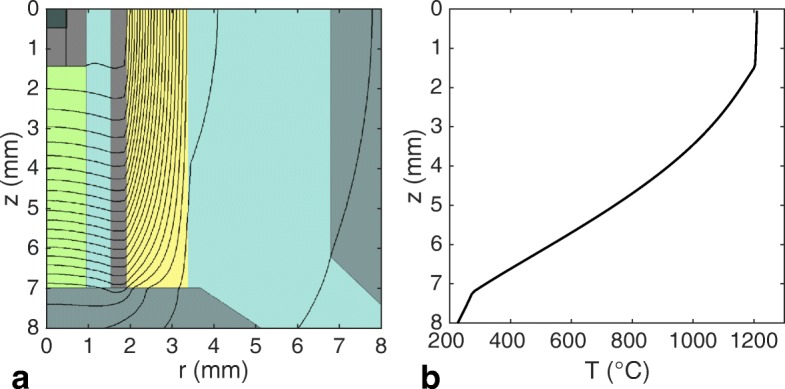
**a** The temperature distribution in the assembly, as modelled with the numerical model of Hernlund et al. [43]. Constant colour areas are indicative of sample (black), graphite (grey), Al_2_O_3_ thermocouple tubing (green), MgO (cyan), Zr_2_O_3_ (yellow) and WC cubes (darkish cyan). Black contours lines represent isotherms separated by 50 degree intervals, with the 1200 °C isotherm located closest to the sample (at z = 1.5 mm and *r* = 0 mm). **b** The modelled temperature profile along the assembly’s axis (the *r* = 0 line)

The results indicate that temperatures interior to the graphite heater vary most strongly in axial direction (Fig. 8). This temperature gradient is small interior to the graphite sample container (z < 1.5 mm), because graphite has a high thermal conductivity. The temperature at the thermocouple junction (at z = 1.5), is only six degrees below the central temperature of 1200 °C according to this numerical model. These modelled variations are consistent with the < 20 °C variations in the central 3 mm of the assembly measured by van Westrenen et al. [44] using spinel growth kinetics in a similar assembly. The modelled temperature drops by 90 °C from z = 1.5 mm to z = 2.5 mm, illustrating the importance of placing the thermocouple junction close to the sample container. In practice, the hard 4-bore Al_2_O_3_ tube holding the thermocouple wires is usually effectively pressing the thermocouple junction onto the graphite bucket while it is pressurized by the WC anvils, which ensures that the junction is usually at or very close to the boundary between the sample container and Al_2_O_3_ tubing. This implies that the temperature read-out is usually fairly accurate, provided that the total length of the sample and container combination is below 3 mm in z-direction.

An additional error on the temperature measurement arises from the pressure effect on the electromotive force induced by the Seebeck effect that is not taken into account (the ASTM E230/E230M standard is calibrated at ambient pressures). This pressure effect on the thermocouple is expected to be small at temperatures below 1500 °C and is universally neglected in high-pressure setups around the world [45].

Table 1 shows that a significant number of experiments experienced thermocouple failure. This is particularly the case for the earlier experiments (numbered MA25 and lower), of which 60% had failed thermocouples. In these early experiments, we used thermocouple wires of 0.018 mm diameter. The later experiments, numbered MA26 and beyond, were performed with slightly thicker thermocouple wires of 0.025 mm thickness, which help prevent thermocouple failures (28% thermocouple failure). We are currently taking additional measures to further reduce thermocouple failures.

**Fig. 9:**
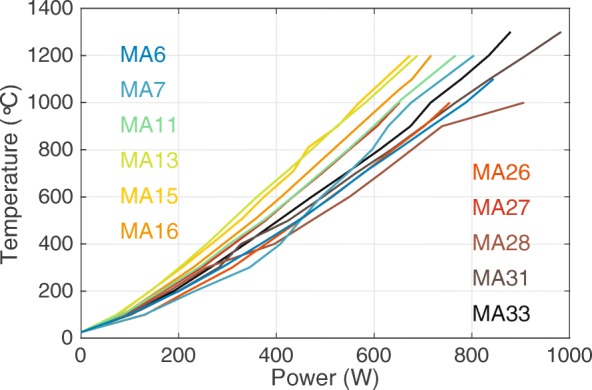
The relation between electrical power and thermocouple temperature as recorded for the experiments in this study (Table 1). Power and temperature are recorded at every subsequent 100 degree interval and linearly interpolated

In experiments where thermocouple readout failed, the sample temperature is estimated by the relation between power and temperature as recorded in comparable experiments with surviving thermocouples (Fig. 9). The power-temperature relation varies per experiment for several reasons. First, while our calibration work progressed, we were increasingly successful in machining thinner graphite heaters which heat more efficiently. This is reflected in Fig. 9 by the lower temperature in the MA6, MA7, and MA11 experiments compared to the MA13, MA15 and MA16 experiments at similar powers. Second, heating is less efficient at higher pressures, which is reflected by the lower temperature in the experiments from MA26 to MA32 at similar power compared to the MA13, MA15 and MA16 experiments. We took these effects into consideration when we estimated the temperature of experiments with failed thermocouples. The resulting errors on these estimates are likely on the order of ±150 degrees. The power used in experiment MA28 is exceptionally high in relation to those of other experiments at similar pressures, in particular in relation to experiment MA23 that was performed at identical pressure. We suspect that the temperature measurement of the MA28 experiment was in error by ~ 200 °C (too low), potentially as a result of a rare displacement of the thermocouple junction away from the sample container. This may have placed MA23 at the garnet-perovskite boundary and the hotter MA28 in the stability field of perovskite.

### Pressure errors

The phase transitions used for the high-temperature quench experiments are relatively insensitive to temperature. We estimate that the temperature error propagates to an error in the pressure calibration of below 0.2 GPa.

The largest error of our pressure calibration originates from uncertainties in the pressure of the phase transitions themselves. These errors accumulate with increasing pressure because fully hydrostatic experiments (when samples pressure can be directly calculated by the amount of applied force) can only be performed at pressures below ~ 3 GPa, as most appropriate liquid pressure media will accumulate stresses at higher pressures (e.g. [22]). A pressure standard for experimental studies for higher pressure phase relations relies on the extrapolation of crystalline behavior at low pressures using thermodynamic formulations (equations of state). The errors on these extrapolations increase with increasing pressure. As a result, the standard error on the phase transitions used in this study are below ±0.1 GPa for the Bi I-II transition, ±0.2 GPa for the quartz-coesite transition in SiO_2_ and the garnet-perovskite transition in CaGeO_3_, ±0.3 GPa for the Bi III-V transition and ± 0.4 GPa for the coesite-stishovite transition in SiO_2_.

Another error originates from the identification of and interpolation between the calibration points. This error is difficult to quantify, but likely does not exceed 0.3 GPa. The total error on the pressure calibration grows from ±0.2 GPa at 100 bars to ±0.6 GPa at 400 bars.

## Conclusions

The 18/11 assembly as calibrated in this study can expose cubic millimeter sized samples to pressure and temperature conditions that exist in deep planetary interiors (down to a depth of ~ 300 km in Earth, ~ 650 km in Mercury and ~ 750 km in Mars). The apparatus can be used to test high-pressure phase relations predicted by ab initio and thermodynamic calculations (e.g. [15, 16]), and to give insights into the origin of surface material that is thought to originate from great depths. These types of experimental constraints on deep planetary matter are complementary to other approaches currently adopted in interior planetary modeling studies ([11–14]). In the future, a smaller 8 mm / 3 mm OEL/TEL assembly can be used in the same press expanding the accessible pressure range to a maximum of ~ 25 GPa (e.g. [21]).

## Abbreviations

Coe: Coesite
Grt: Garnet
OEL: Octahedral edge length
Prv: Perovskite
Qtz: Quartz
Stv: Stishovite
TEL: Truncated edge length
